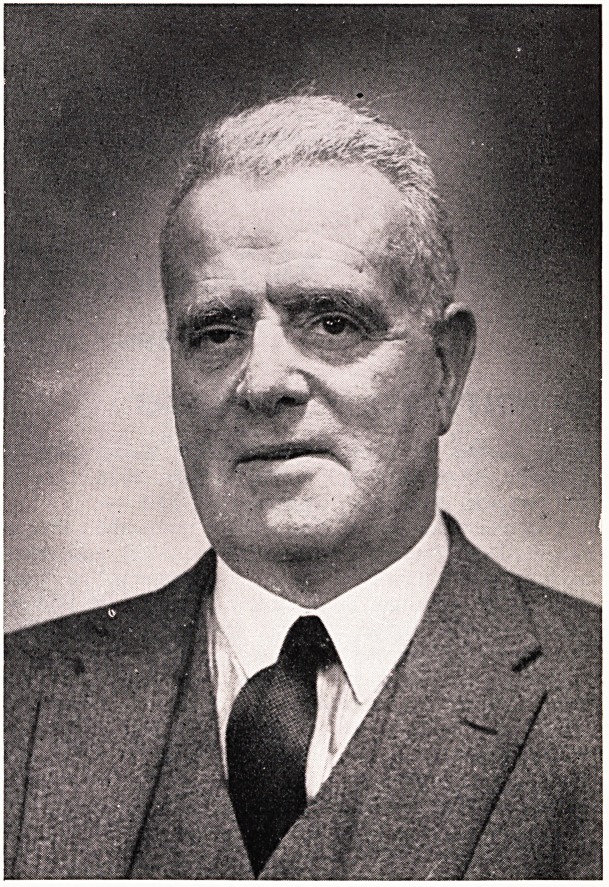# G. L. Alexander

**Published:** 1971-04

**Authors:** 


### Obituary

### G. L. ALEXANDER
B.Sc., M.D., F.R.C.S., F.R.C.S.E.

George Lionel Alexander, former head of the Regional Neurosurgical Unit at Frenchay Hospital, Bristol, died after three and a half years of retirement at his home at Painswick in the Costwolds on October 8th, 1970, at the age of 68 years. He was a man of outstanding personality and achievement, whose talents had been devoted to his chosen speciality since 1933, when he joined the Department of Neurological Surgery founded in Edinburgh by Professor Norman Dott, who was with Sir Geoffrey Jefferson and Sir Hugh Cairns, one of the triumvirate that constituted British neurosurgery in those years. George Alexander was appointed to the honorary staff of the Edinburgh Royal Infirmary three years later, and like many others in the days before the National Health Service, devoted his unflagging energies through long days and nights in a particularly arduous calling to the public service for the sick, with meagre rewards in private practice.

In 1938 he spent a year at Chicago on a Rockefeller Scholarship, in research in neuro-anatomy and physiology. Never one to neglect opportunities, he had the good fortune to meet his future wife on the voyage home, and they were married shortly after the outbreak of war.

During the war he carried an enormous load in an expanded department and a growing speciality at the Edinburgh Royal Infirmary and Bangour Hospital, but found time to develop special interests in treatment and rehabilitation of head injuries, intracranial aneurysms and spinal surgery. In this last, he initiated an antero-lateral approach for surgical treatment of tuberculous spines.

In 1948 he was appointed Director of the new University Neurosurgical Unit in Bristol, with beds at Frenchay Hospital, and soon to be incorporated in the N.H.S. as a Regional Unit which served the whole of the South West until the establishment of the Unit at Plymouth in November 1964. In addition to great attention to the details of organisation of his new department, he had the capacity and foresight personally to design and get passed by the Ministry of Works, before the long frustrations of the N.H.S. and enforced parsimony of the Ministry of Health, plans for a new theatre block at Frenchay. This was on a generous scale with full air conditioning. as good as any in the country. It was opened, along with an adjacent wartime "block" converted to neuroradiological department, examination rooms, and offices, by Sir Geoffrey Jefferson in 1953. These premises immediately adjacent to the adult wards for the specialty, contributed enormously to the building up of a highly efficient department.

George Alexander set an example of unflagging maintenance of high standard and devotion to duty at all times. He inspired loyalty in all his staff, and many now in independent practice look back with gratitude, respect and affection for their old "chief". Bristol and the South West owed him much for valuable service given without stint over twenty years.

He continued to develop and encourage advances in neurosurgery. He travelled to clinics and conferences at home and abroad, was a member of the Surgical Traveller's Club, and a corresponding member of the Scandinavian, French, Portuguese and Spanish Neurosurgical Societies. He was a Patterson-Smythe lecturer in Montreal in 1956, and a Honyman Gillespie lecturer in Montreal in 1957. From 1964 to 1966 he was President of the Society of British Neurological Surgeons. In conjunction with the late Dr. R. M. Norman, he published an outstanding monograph on the Sturge-

Weber syndrome. In this he advocated early surgical removal of angiomatus portions of brain before resulting status epilepticus in infancy has produced crippling effects. In this as in other fields, he continued to work for understanding and advances in practice. His advice was sought and much time spent in hospital committees and professional organisation.

George's professional commitments always took first call on his time and abundant energies, leaving very little for leisure pursuits, though he was a good shot, a keen fisherman, and intrepid sailor. Only when he retired, it seemed, could he devote all the time they deserved to his family and his own garden, filled with roses. Now the traditional stern Scottish facade was mellowed, like his Cotswold stone walls. Many friends and colleagues gathered to pay their last respects in the Autumn sunshine at Painswick Church, with living memories of a happy man, his life fulfilled in service to others.

## Figures and Tables

**Figure f1:**